# ENPP1 enzyme replacement therapy improves ectopic calcification but does not rescue skeletal phenotype in a mouse model for craniometaphyseal dysplasia

**DOI:** 10.1093/jbmrpl/ziae103

**Published:** 2024-08-08

**Authors:** Ernst J Reichenberger, Kevin O’Brien, Ayano Hatori, Thomas O Carpenter, Koen van de Wetering, Lisa Flaman, Jennifer Howe, Daniel Ortiz, Yves Sabbagh, I-Ping Chen

**Affiliations:** Center for Regenerative Medicine and Skeletal Development, Department of Reconstructive Sciences, School of Dental Medicine, University of Connecticut Health, Farmington, CT 06030, United States; Research and Development, Inozyme Pharma, Boston, MA 02210, United States; Department of Endodontology, School of Dental Medicine, University of Connecticut Health, Farmington, CT 06030, United States; Department of Pediatrics (Endocrinology), Yale University School of Medicine, New Haven, CT 06520, United States; Department of Dermatology and Cutaneous Biology, Jefferson Institute of Molecular Medicine and PXE International Center of Excellence in Research and Clinical Care, Sidney Kimmel Medical College, Thomas Jefferson University, Philadelphia, PA 19144, United States; Research and Development, Inozyme Pharma, Boston, MA 02210, United States; Research and Development, Inozyme Pharma, Boston, MA 02210, United States; Research and Development, Inozyme Pharma, Boston, MA 02210, United States; Research and Development, Inozyme Pharma, Boston, MA 02210, United States; Center for Regenerative Medicine and Skeletal Development, Department of Reconstructive Sciences, School of Dental Medicine, University of Connecticut Health, Farmington, CT 06030, United States; Department of Endodontology, School of Dental Medicine, University of Connecticut Health, Farmington, CT 06030, United States

**Keywords:** bone QCT/μCT, genetic animal models, osteoblasts, osteoclasts

## Abstract

Craniometaphyseal dysplasia (CMD) is a rare genetic bone disorder, characterized by progressive thickening of craniofacial bones and flared metaphyses of long bones. Craniofacial hyperostosis leads to the obstruction of neural foramina and neurological symptoms such as facial palsy, blindness, deafness, or severe headache. Mutations in *ANKH* (mouse ortholog *ANK*), a transporter of small molecules such as citrate and ATP, are responsible for autosomal dominant CMD. Knock-in (KI) mice carrying an ANK_F377del_ mutation (*Ank^KI/KI^*) replicate many features of human CMD. Pyrophosphate (PPi) levels in plasma are significantly reduced in *Ank^KI/KI^* mice. PPi is a potent inhibitor of mineralization. To examine the extent to which restoration of circulating PPi levels may prevent the development of a CMD-like phenotype, we treated *Ank^KI/KI^* mice with the recombinant human ENPP1-Fc protein IMA2a. ENPP1 hydrolyzes ATP into AMP and PPi. Male and female *Ank^+/+^* and *Ank^KI/KI^* mice (*n* ≥ 6/group) were subcutaneously injected with IMA2a or vehicle weekly for 12 wk, starting at the age of 1 wk. Plasma ENPP1 activity significantly increased in *Ank^KI/KI^* mice injected with IMA2a (Vehicle/IMA2a: 28.15 ± 1.65/482.7 ± 331.2 mOD/min; *p* <.01), which resulted in the successful restoration of plasma PPi levels (*Ank^+/+^*/*Ank^KI/KI^* vehicle treatment/*Ank^KI/KI^* IMA2a: 0.94 ± 0.5/0.43 ± 0.2/1.29 ± 0.8 μM; *p* <.01). We examined the skeletal phenotype by X-Ray imaging and μCT. IMA2a treatment of *Ank^KI/KI^* mice did not significantly correct CMD features such as the abnormal shape of femurs, increased bone mass of mandibles, hyperostotic craniofacial bones, or the narrowed foramen magnum. However, μCT imaging showed ectopic calcification near basioccipital bones at the level of the foramen magnum and on joints of *Ank^KI/KI^* mice. Interestingly, IMA2a treatment significantly reduced the volume of calcified nodules at both sites. Our data demonstrate that IMA2a is sufficient to restore plasma PPi levels and reduce ectopic calcification but fails to rescue skeletal abnormalities in *Ank^KI/KI^* mice under our treatment conditions.

## Introduction

The rare genetic bone disorder craniometaphyseal dysplasia (CMD) is characterized by progressive thickening of craniofacial bones and flaring metaphyses of long bones.^1^ Patients with CMD often suffer from neurological symptoms, including severe headache, hearing loss, visual impairment, and facial palsy.^2^^,^^3^ CMD can be diagnosed early in infants based on clinical, radiographic, and genetic findings. Hyperostosis of craniofacial bones frequently lead to the obstruction of cranial foramina and nerve compression.^4^ These debilitating symptoms progress throughout life. CMD can be inherited as autosomal dominant (AD) and autosomal recessive (AR) traits.^5^ Mutations in the progressive ankylosis gene (*ANKH*) are responsible for AD CMD and a mutation in the gap junction protein alpha 1 (*GJA1*) causes AR CMD.^6-8^ AD CMD occurs more frequently than AR CMD. The expressivity of CMD can vary even within the same family.^9^ To date, treatment for CMD is limited to surgical decompression of obstructed foramina and plastic surgery for correcting craniofacial structures.^10^

ANKH (mouse ortholog ANK) is highly conserved within vertebrates and ubiquitously expressed in skeletal and non-skeletal tissues. ANK protein is localized on plasma membranes, endoplasmic reticulum, clathrin-coated vesicles, and the Golgi apparatus.^11^^,^^12^ The function of ANK depends on its localization. In cellular compartments, ANK is required for endosomal function and Golgi-endosomal membrane trafficking.^12^ ANK interacts with clathrin and clathrin-associated adaptor protein complexes and regulates tubular carrier formation in the trans-Golgi network.^12^ Historically, ANK/ANKH has been thought to transport intracellular PPi into the extracellular matrix to regulate or prevent tissue mineralization.^13^^,^^14^ Recent studies, however, demonstrated that ANK/ANKH is involved in the release of other small molecules, including citrate and ATP.^15^^,^^16^ Inhibition of ANK results in decreased extracellular ATP (eATP) levels, which has been shown in primary articular chondrocyte cultures.^17^ ANK is a positive regulator of osteoblastic and osteoclastic differentiation.^18^^,^^19^ ANK loss of function accelerates chondrocyte maturation and increases adipogenic differentiation in *Ank^ank/ank^* mice.^20^^,^^21^

To study the pathogenesis of CMD, we generated a knock-in (KI) mouse model carrying an in-frame deletion of phenylalanine 377 (F377del) in ANK. *Ank^KI/KI^* mice replicate many features of human CMD, including hyperostotic craniofacial bones, narrowed cranial foramina, and abnormal shape of femurs with extensive diaphyseal trabeculation.^22^ Ectopic mineralization at multiple sites including joints has been observed in *Ank^KI/KI^* mice,^22^ although this is not typical for human CMD. Consistent with data from CMD patients, serum alkaline phosphatase levels are significantly higher. PTH, 25(OH) vitamin D, intact FGF23, and the C-terminal forms of FGF23 are within normal range in *Ank^KI/KI^* mice.^22-24^ Adult *Ank^KI/KI^* mice have normal serum Pi but significantly reduced plasma PPi levels.^22^^,^^23^ In osteoclast cultures derived from *Ank^KI/KI^* mice and CMD patients, we showed that osteoclast formation and function are reduced.^25^^,^^26^ We found deficient mineral nodule formation in *Ank^KI/KI^* osteoblast cultures, although dynamic histomorphometry of femoral metaphyses was comparable between *Ank^+/+^* and *Ank^KI/KI^* mice.^22^^,^^25^ Taken together, ANK/ANKH mutations in CMD affect both osteoblast and osteoclast function and result in an imbalanced Pi/PPi ratio.

Imbalanced Pi/PPi ratios have been associated with mineral-related pathological conditions.^27^^,^^28^ Pi is a major component of hydroxyapatite and PPi is an inhibitor of mineralization.^29^ Extracellular PPi (ePPi) in the skeleton is co-regulated by ectonucleotide pyrophosphatase /phosphodiesterase-1 (ENPP1), tissue-nonspecific alkaline phosphatase (TNAP), and ANK/ANKH.^29^ ENPP1 hydrolyzes ATP to AMP and PPi, and TNAP hydrolyzes ePPi to generate Pi. A disturbed PPi/P ratio can result in ectopic calcifications, which is a shared phenotype of Enpp1-deficient tiptoe walking (ttw/ttw) mice, *Enpp1^asj/asj^* mice with a point mutation in *Enpp1*, *ENPP1^asj-2J/asj-2J^* mice with a large deletion/insertion in the ENPP1 gene, *Ank^ank/ank^* mice, and *Ank* knockout (*Ank^KO/KO^*) mice.^13^^,^^27^^,^^30-33^ ENPP1 enzyme supplementation successfully decreases mortality, restores circulating levels of PPi, and prevents pathological calcification in ENPP1 loss-of-function mouse models for generalized arterial calcification of infancy (GACI).^33-35^

Here, we examine whether correction of reduced plasma PPi can rescue the CMD-like skeletal phenotype in *Ank^KI/KI^* mice. We dosed male and female mice with vehicle or with recombinant human Enpp1-Fc protein, IMA2a, for 12 wk. These findings may have clinical implications for treating patients with CMD.

## Materials and methods

### Mouse model

A mouse model for AD CMD had previously been generated by introducing a TTC_1130-1132_ (phenylalanine 377) deletion in exon 9 of the *Ank* gene.^22^ Mice were generated in 129/Sv background and then backcrossed with C57Bl/J6 mice (N8). Mouse genotype and sex were determined at 1 wk after birth as previously described.^22^^,^^36^ Primer sequences for PCR genotyping are listed in Table S1. The *Ank* genotype was re-confirmed at the time of sacrificing animals. Animal protocol (AP-200644-0225) was approved by the Institutional Animal Care and Use Committee of the University of Connecticut Health, and all work was performed in an AAALAC accredited facility.

### ENPP1-fc (IMA2a)

The IMA2a fusion protein is comprised of the extracellular portion of human ENPP1, which is linked to the N-terminus of a human IgG1 Fc domain. IMA2a pharmacokinetics in mice were optimized by inclusion of YTE mutations^37^ in the Fc portion and by creation of an N-linked glycosylation site in the catalytic domain of ENPP1. The IMA2a fusion protein was produced in CHO cells by fed-batch cell culture and was purified using protein A affinity chromatography.

### ENPP1-fc (IMA2a) dosing

Mice were injected once a week either with vehicle (2.5 mL/kg) or with IMA2a (5 mg/kg; 2.5 mL/kg) (Inozyme Pharma, Inc., Boston, MA) formulated in vehicle, beginning at the age of day 7 for a period of 12 wk. The vehicle was composed of 1× PBS. In addition to IMA2a, mice received GK1.5 (BioXCell, Lebanon, NH) to block CD4-mediated cell adhesion and T cell activation for preventing immune reactions against the human IMA2a protein. Mice were dosed with 40 μg GK1.5 per animal on day 6 after birth and thereafter with 25 μg per animal on the day of IMA2a injections. GK1.5 was injected subcutaneously and IMA2a intraperitoneally.

### Measurement of plasma PPi level and ENPP1 activity

Plasma samples were collected from the submandibular vein of Ank+/+ and AnkKI/KI mice using an animal lancet (Goldenrod, Braintree Scientific, MA) at the endpoint of the experiment (12 wk after the first IMA2a injection). Mouse blood was collected into lithium heparin tubes (Sarstedt, Numbrecht, Germany) and immediately centrifuged at 5000 rpm for 5 min at 4 °C. Supernatant (125 μL) was transferred to an Amicon Ultra 50KD MWCO 0.5 mL filter tube (Sartorius, Gottingen, Germany) and the filtrate was stored at −80 °C until further processing, and 25 μL of unfiltered plasma was used for ENPP1 activity determination.

Plasma PPi was measured by a luminescent assay, adapted from Jansen et al. and Khan et al.^33^^,^^38^ PPi was first converted to ATP by ATP sulfurylase in the presence of adenosine 5´-phosphosulfate (APS) and ATP levels were then measured by a luminescent assay to obtain a total luminescence signal. A standard curve was created by spiking with known concentrations of PPi (Santa Cruz Biotechnology, Dallas, TX) in water, and 15 μL of filtered plasma samples were mixed with 5 μL of assay buffer (40 mM HEPES pH 7.4, 8 mM CaCl_2_, 2 mM MgSO_4_) containing 16 μM adenosine 5´-phosphosulfate (Sigma-Aldrich, St. Louis, MO), 0.1 U/mL ATP sulfurylase (R&D Systems, Minneapolis, MN). Reactions were incubated at 37 °C for 40 min, followed by 10 min at 90 °C to deactivate the ATP sulfurylase. Resulting ATP was then quantified by mixing the reaction mixture in a 1:1 ratio with BactiterGlo detection reagent (Promega, Madison, WI). To account for endogenous ATP present in the plasma, a blank reaction was run for each sample with heat-inactivated ATP sulfurylase. The luminescence signals from this reaction were subtracted from the total luminescence signals to calculate plasma PPi levels.

ENPP1 activity was measured by a colorimetric assay adapted from Jansen et al.^39^ Samples were diluted as needed in mouse plasma (BioIVT, Westbury, NY), and 10 μL of diluted samples were incubated at 37 °C with 90 μL of assay buffer (1 M Tris pH 8.0, 50 mM NaCl, 20 μM CaCl_2_, 20 μM ZnCl_2_) containing 2 mM pNP-TMP (Sigma-Aldrich, St. Louis, MO). The production of p-nitrophenol was kinetically analyzed by measuring the absorbance at 405 nm using a SPECTRAmax Plus 384 microplate reader (Molecular Devices, San Jose, CA). The activity was calculated as the change in absorbance over time (mOD/min).

### Skeletal analysis

For skeletal analysis, we collected 6-8 male and female *Ank^+/+^* and *Ank^KI/KI^* mice dosed with vehicle or IMA2a. Body weight was measured weekly and mice were sacrificed at 13 wk of age. Skulls, mandibles, femurs, and feet were dissected and fixed in 4% paraformaldehyde and subjected to X-ray imaging (Kubtec Scientific, Stratford, CT) and computed microtomography (μCT) in the MicroCT facility at UConn Health (ScanCo Medical AG, Bassersdorf, Switzerland). Radiographic opacity was determined by the intensity for gray value, in regions that included nasal bone and tympanic bullae, using histograms in Adobe Photoshop (Figure S1A). We measured these regions because CMD patients have excessive bone mass in these regions. Mandibular bone mass was determined by measuring the expanded surface below the lower incisor to the distal of third molars (Figure S1B). For micro-CT study, total volume (TV) and bone volume (BV) of mandibles were calculated by measuring vertical sections from mesially of first molars to distally of second molars. Trabecular measurements and cortical bone parameters of femurs were obtained as previously described.^22^ Briefly, trabecular measurements of femurs were taken at the distal growth plate in 80 consecutive slices of 12 μm resolution over a distance of 960 μm. Volumetric regions were rendered as 3D arrays with an isometric voxel dimension of 12 μm, and 50 cross-sectional slices of 12 μm in the mid-diaphysis were used to calculate cortical bone parameters.

BV of ectopic mineralization in skulls was determined by adding areas of serial μCT images through entire nodules (Figure S2). Cross-sectional slices of joints were taken 450 μm from the base of metacarpal bones and volumetric toe calcifications were determined by adding ectopic calcifications from 20 consecutive slices of 12 μm resolution over a distance of 240 μm. The widths and lengths of foramina magna were measured by Fiji Image J software (NIH).

### Statistical analysis

**Figure 1:**
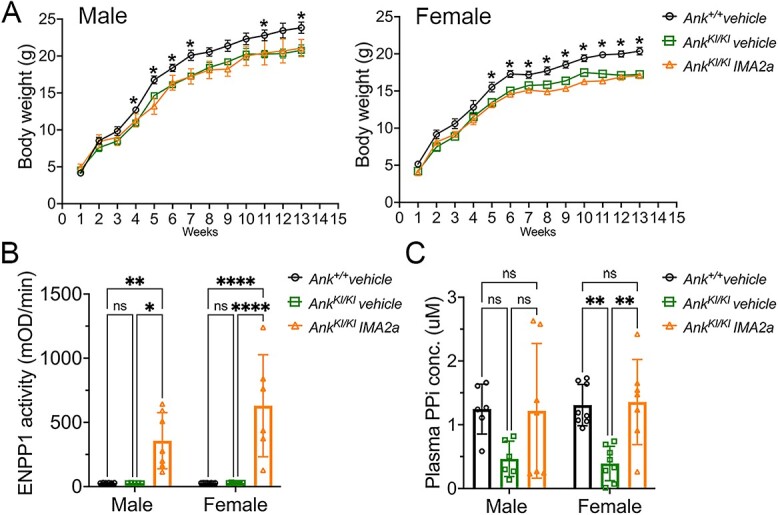
Changes in body weight, ENPP1 activity, and plasma PPi levels in *Ank^KI/KI^* mice after IMA2a injection. (A) *Ank^KI/KI^* mice dosed with vehicle or IMA2a gained less weight compared with *Ank^+/+^* mice between weeks 1 and 13; (B) IMA2a injection increased ENPP1 activity in plasma; (C) IMA2a injection reversed the overall trend of reduced plasma PPi levels in *Ank^KI/KI^* mice. *p <.05 and **p <.01 indicate statistical significance by two-way ANOVA with Tukey’s multiple comparison test; ns: no significant difference.

**Figure 2:**
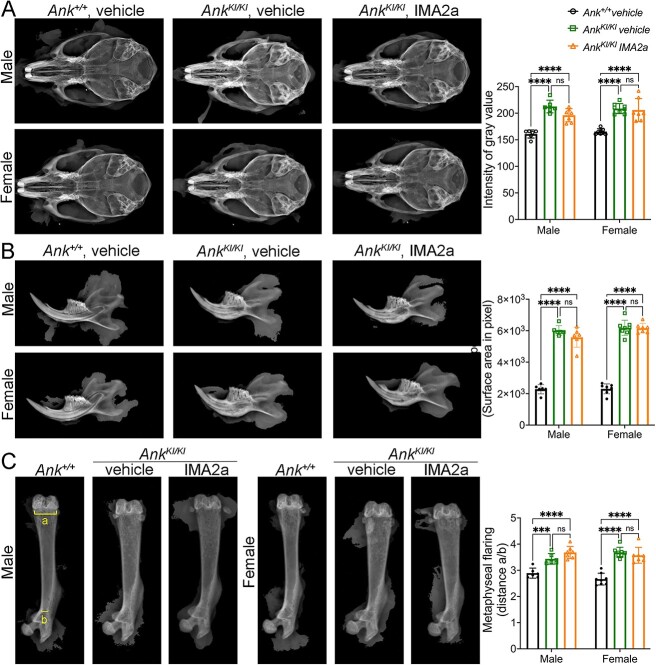
Representative radiographic images of skeletal phenotypes in male and female *Ank*^+/+^ and *Ank*^KI/KI^ mice. *Ank^KI/KI^* mice with IMA2a treatment retained (A) increased radiopacity of skulls; (B) increased bone surface area of mandibles; and (C) widened metaphyses as determined by the ratio of distances a and b (a/b) in femurs (indicated by yellow brackets). *p <.05, **p <.01, and *** or ****p <.001 indicate statistical significance by two-way ANOVA with Tukey’s multiple comparison test; ns: no significant difference.

**Figure 3:**
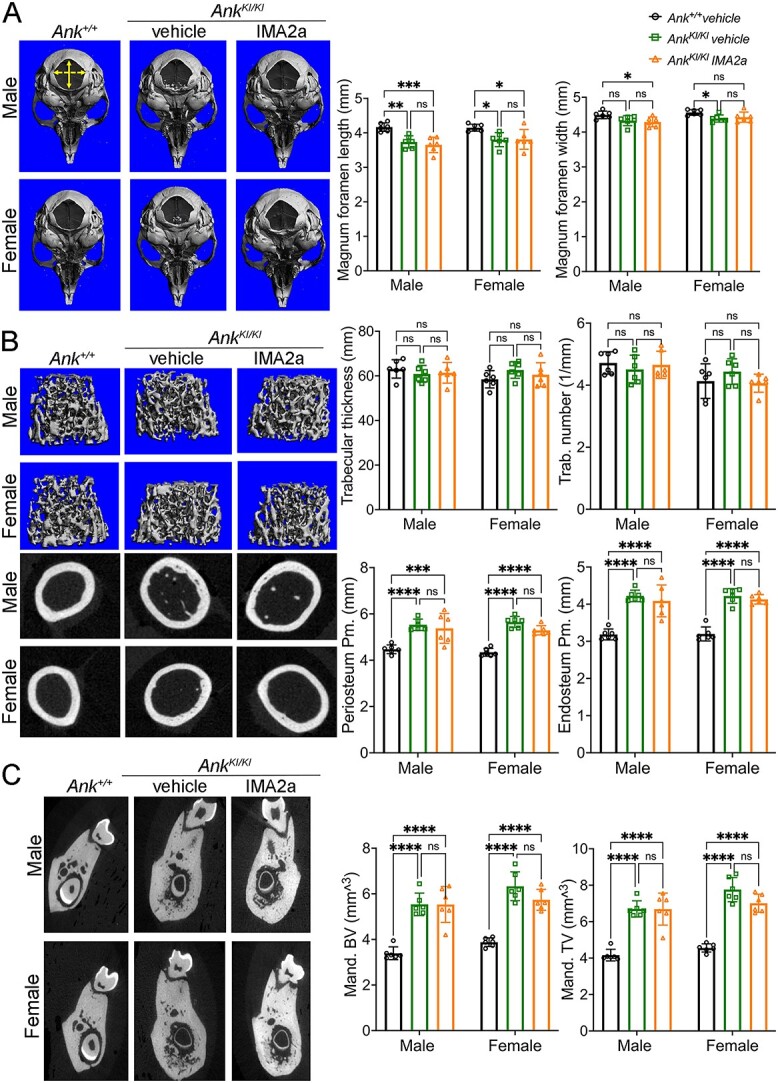
μCT analysis of CMD-like skeletal phenotypes in *Ank^KI/KI^* male and female mice with or without IMA2a treatment. (A) Representative images of 3D reconstruction of skulls (left panel) and quantitative data for length (solid yellow arrow) and width (dashed yellow arrow) (right panel) of foramina magna; (B) 3D reconstruction of trabeculation in metaphyses and cross-sectional slices of cortical bone in diaphyses, and quantitative data of trabecular and cortical parameters; (C) cross-sectional slices through furcation of first molars and quantitative data. Trab.: trabecular; Pm: perimeter; BV: bone volume; TV: total volume. Statistical analyses performed by two-way ANOVA with Tukey’s multiple comparison test.

Statistical analysis was performed by Student’s *t*-test, or two-way ANOVA followed by Tukey’s multiple comparison test, using Prism 5 software (GraphPad Software, La Jolla, CA). Results of two-way ANOVA, including effects of grouping, sex, and interaction, used in Figures 1-3 are summarized in Table S2.

## Results

### Effects of IMA2a on ENPP1 activity and PPi levels in *Ank*^*KI/KI*^ mice

To examine the effects of IMA2a on *Ank^KI/KI^* mice, we dosed 1-wk-old male and female mice (*n* = 6-8) weekly with vehicle or IMA2a together with GK 1.5 for 12 wk. We previously reported that *Ank^KI/KI^* mice gain less weight in comparison to their *Ank^+/+^* littermates.^22^ We measured the body weight of male and female *Ank^+/+^* and *Ank^KI/KI^* groups injected with vehicle or IMA2a weekly from 1 to 13 wk. IMA2a did not improve weight gain in either male or female *Ank^KI/KI^* mice (Figure 1A).

To evaluate the mouse response to IMA2a, we measured the ENPP1 activity as well as PPi levels in plasma at the endpoint of 13 wk of age. ENPP1 activity was not significantly different from *Ank^+/+^* and *Ank^KI/KI^* mice that received vehicle treatment (Figure 1B). As expected, *Ank^KI/KI^* mice injected with IMA2a exhibited significantly higher ENPP1 activity in plasma (Figure 1B). However, plasma PPi levels were significantly reduced in female *Ank^KI/KI^* mice treated with vehicle (Figure 1C) when compared with *Ank^+/+^* mice and showed a trend of reduction in male *Ank^KI/KI^* mice (male *Ank^+/+^* vehicle vs male *Ank^KI/KI^* vehicle, *p* =.0597). IMA2a injection successfully elevated plasma PPi levels to wild-type levels in *Ank^KI/KI^* mice (Figure 1C).

### Effects of IMA2a on CMD-like skeletal phenotypes in *Ank*^*KI/KI*^ mice

First, we analyzed the skeletal phenotype by Kubtec X-ray imaging. In general, IMA2a treatment did not significantly prevent the abnormal radiographic features, including increased radiopacity of skulls (Figure 2A), hyperostotic mandibles (Figure 2B), and flaring metaphyses of femurs (Figure 2C) observed in both male and female *Ank^KI/KI^* mice.

We next analyzed skulls, femurs, and mandibles of *Ank^+/+^* and *Ank^KI/KI^* mice by μCT. *Ank^KI/KI^* mice have narrowed foramina magna (measured by length and width), when compared with *Ank^+/+^* mice (Figure 3A).^22^ IMA2a treatment did not rescue the smaller foramina magna of *Ank^KI/KI^* mice (Figure 3A). In femurs, we measured metaphyseal trabeculation and diaphyseal (mid-shaft of femurs) cortical parameters and showed that there was no significant difference between *Ank^KI/K^* mice treated with vehicle or IMA2a (Figure 3 and Table 1). The cortical thickness in *Ank^KI/KI^* mice was significantly less and cortical porosity was significantly higher when compared with *Ank^+/+^* mice (Table 1). *Ank^KI/KI^* mice have enlarged periosteal and endosteal diameters, which were not changed by IMA2a treatment (Figure 3B).^22^ IMA2a reversed the trend of certain femoral parameters, although not statistically significant, such as BV/TV, trabecular thickness, trabecular number, and trabecular spacing, especially in female *Ank^KI/KI^* mice (Figure 3 and Table 1). Lastly, the BV and TV of mandibles of male and female *Ank^KI/KI^* mice remained significantly increased after vehicle or IMA2a treatment when compared with *Ank^+/+^* mice (Figure 3C).

**Table 1:** Micro-CT measurements from male and female *Ank^+/+^* mice treated with vehicle and *Ank^KI/KI^* mice treated with vehicle or IMA2a.

Parameters	Male			Female		
**Femur trab.**	*Ank^+/+^, vehicle*	*Ank^KI/KI^, vehicle*	*Ank^KI/KI^, IMA2a*	*Ank^+/+^, vehicle*	*Ank^KI/KI^, vehicle*	*Ank^KI/KI^, IMA2a*
**BV (mm^3^)**	0.48 ± 0.08	0.52 ± 0.16	0.51 ± 0.16	0.37 ± 0.09^a^	0.56 ± 0.11^a^	0.46 ± 0.12
**TV (mm^3^)**	2.40 ± 0.19^a,b^	3.18 ± 0.15^a^	2.93 ± 0.37^b^	2.42 ± 0.14^a,b^	3.18 ± 0.22^a^	3.14 ± 0.15^b^
**BV/TV (%)**	20.21 ± 3.38	16.33 ± 4.59	17.58 ± 5.00	15.65 ± 4.22	17.88 ± 4.46	14.75 ± 4.19
**Tb. Sp (mm)**	208.2 ± 17.5	213.5 ± 25.7	204.3 ± 24.1	241.4 ± 38.3	215.3 ± 25.6	237.5 ± 23.0
**BMD (mg/ccm HA)**	202.71 ± 19.5	165.44 ± 34.9	178.75 ± 33.2	176.63 ± 24.1	187.15 ± 27.7	167.37 ± 29.1
**TMD (mg/ccm HA)**	800.81 ± 26.0	810.03 ± 9.8	816.51 ± 18.0	798.24 ± 16.6	822.40 ± 10.0	819.52 ± 24.4
**Femur cort.**	*Ank^+/+^, veh.*	*Ank^KI/KI^, veh*	*Ank^KI/KI^, IMA2a*	*Ank^+/+^, veh.*	*Ank^KI/KI^, veh*	*Ank^KI/KI^, IMA2a*
**Tt. Ar (mm^^2^)**	1.58 ± 0.12^a,b^	2.24 ± 0.12^a^	2.07 ± 0.36^b^	1.52 ± 0.11^a,b^	2.30 ± 0.19^a^	2.15 ± 0.07^b^
**Ct. Ar (mm^^2^)**	0.79 ± 0.08	0.88 ± 0.07	0.80 ± 0.12	0.71 ± 0.04^a,b^	0.93 ± 0.05^a^	0.83 ± 0.03^b^
**Ct. Th (mm)**	0.20 ± 0.01^a,b^	0.18 ± 0.01^a^	0.16 ± 0.01^b^	0.18 ± 0.01	0.18 ± 0.005	0.17 ± 0.007
**Ct. Po (%)**	0.29 ± 0.02^a,b^	0.52 ± 0.13^a^	0.45 ± 0.12^b^	0.32 ± 0.04^a,b^	0.41 ± 0.12^a^	0.43 ± 0.10^b^
**BMD (mg/ccm HA)**	1213.2 ± 9.2^a,b^	1167.8 ± 13.3^a^	1174.1 ± 12.8^b^	1232.61 ± 7.2^a,b^	1190.32 ± 5.6^a,c^	1200.59 ± 4.13^b,c^
**TMD (mg/ccm HA)**	1294.5 ± 11.6^a,b^	1248.59 ± 15^a^	1260.1 ± 12.8^b^	1324.69 ± 13^a,b^	1269.41 ± 7.9^a,c^	1290.31 ± 6.7^b,c^

Abbreviations: BV: bone volume; TV: total volume; Tb. Sp: trabecular spacing; Tt. Ar: total area; Ct. Ar: cortical area; Ct. Th: cortical thickness; Ct. Po: cortical porosity; TMD: tissue mineral density.

a–c (*p* <.05) indicated statistically significant differences between two groups. Statistical analyses performed by one-way ANOVA followed by Tukey’s post-hoc test.

### Effects of IMA2a on ectopic calcification in* Ank*^*KI/KI*^ mice

**Figure 4:**
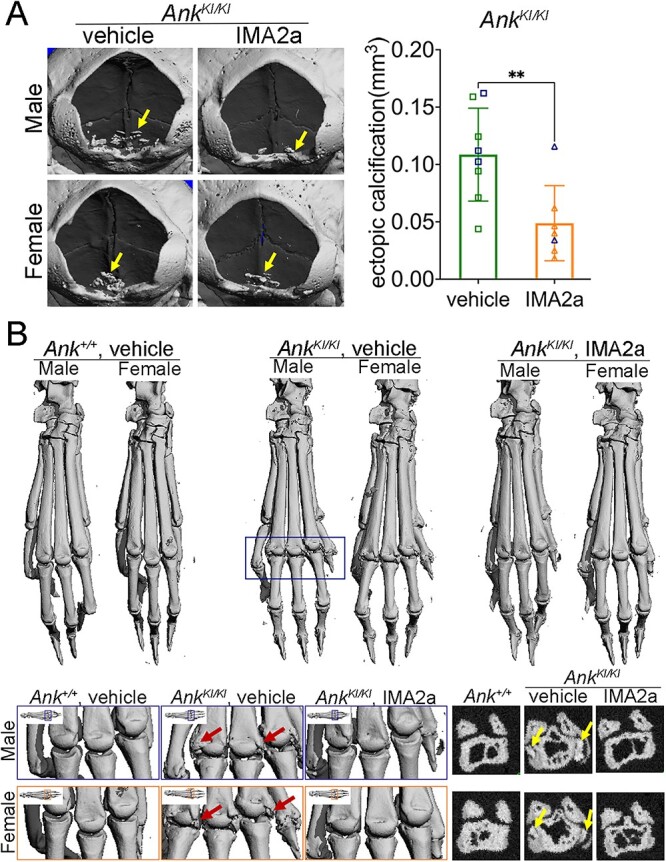
Effect of IMA2a on ectopic calcification in *Ank^KI/KI^* mice. Representative micro-CT images showing IMA2a treatment reduced ectopic calcification (A) nearby the foramen magnum (indicated by yellow arrows) in *Ank^KI/KI^* mice. Graph: Blue data points: size of calcification in male *Ank^KI/KI^* mice; green/ tangerine data points: size of calcification in female *Ank^KI/KI^* mice. (B) Ectopic calcification at foot joints in male and female *Ank^KI/KI^* mice. Magnified images (bottom panels) showing joints between metacarpal and phalangeal bones (blue and orange boxes of the left panel) and cross-sectional slices of the fourth digits (450 μm from the base of metacarpal bones, bottom right panel). Red and yellow arrows indicate ectopic mineral deposition.

ENPP1 enzyme replacement has been reported to rescue intracardiac, aorta, and vibrissae calcifications and reduced plasma PPi in the ENPP1-deficient GACI mouse model.^33-35^ Although not reported in CMD patients, we observed ectopic calcification in *Ank^KI/KI^* mice leading to joint stiffness.^22^ We also noticed the deposition of mineral nodules attached to the meninges near the basioccipital bone at the level of the foramen magnum of *Ank^KI/KI^* mice (Figure S2). We analyzed 12 mice (6 male and 6 female mice) in each group by μCT. Visible calcification in skulls was noted in a total of 8 mice (3 male and 5 female) in the *Ank^KI/KI^* vehicle group and 7 mice (2 male and 5 female) in the *Ank^KI/KI^* IMA2a group. While the number of mice with nodules was not significantly different between the *Ank^KI/KI^* vehicle and *Ank^KI/KI^* IMA2a groups, the BV of ectopic calcifications was significantly less than in *Ank^KI/KI^* mice that were dosed with IMA2a (Figure 4A). *Ank^KI/KI^* mice develop stiffness of joints and reduced ability to grab cage bars approximately at 5 wk of age.^22^ Interestingly, the ectopic mineral deposition of foot joints was visibly less in *Ank^KI/KI^* mice after IMA2a treatment (volume of ectopic calcifications in untreated *Ank^KI/KI^* vs IMA2a treated *Ank^KI/KI^* mice = 0.033 ± 0.006 mm^3^: 0.002 ± 0.002 mm^3^, *p* <.001) (Figure 4B). These bony nodules were mostly deposited close to the bone surface separated from bone via tendon-like tissues rather than an expansion of the bone (Figure 4B). Taken together, these results suggest that IMA2a is sufficient to restore plasma PPi levels and reduce ectopic calcification but does not rescue the CMD-like skeletal abnormalities in *Ank^KI/KI^* mice using this treatment regimen.

## Discussion

CMD was first described in 1954, but ANKH mutations in CMD were not identified until 2001.^1^^,^^6^^,^^7^ Of about 170 publications on CMD in PubMed, most of them are clinical reports and very few involve cellular or molecular research. To date, the pathogenesis of CMD is not fully understood. CMD alleles have been proposed to act as ANKH loss-of-function mutations based on findings that *Ank^KO/KO^* mice recapitulate some CMD features, including thicker skull, narrowed foramen magnum, and fused middle-ear bones.^14^^,^^27^*Ank^KO/KO^* mice, however, do not fully replicate the human CMD phenotype. *Ank^KI/KI^* mice that carry a CMD mutation replicate more characteristic features of CMD than *Ank^KO/KO^* mice. Widened metaphyses of femurs, a main feature in CMD, is only observed in *Ank^KI/KI^* mice.^22^ Unlike *Ank^KO/KO^* mice with no ANK expressed, *Ank^KI/KI^* mice show detectable but low abundance of mutant ANK due to rapid protein degradation.^11^ We proposed that the shared CMD-like phenotype in *Ank^KI/KI^* and *Ank^KO/KO^* mice is caused by loss of a specific function of small molecule transport by ANK and that unique features in *Ank^KI/KI^* mice are contributed by a novel dominant function of mutant ANK. Although a loss-of-function hypothesis alone cannot fully explain the CMD pathogenesis, we hypothesized that correcting reduced plasma PPi levels may partially rescue some CMD-like phenotypes, especially the ones shared between *Ank^KO/KO^* mice and *Ank^KI/KI^* mice. Although here we could show significantly reduced plasma PPi in female *Ank^KI/KI^* mice only, our previous publication reports that plasma PPi levels were also significantly lower in male *Ank^KI/KI^* mice.^23^ The trend of decreased plasma PPi levels that did not reach significance levels in male *Ank^KI/KI^* mice in this study could be due to a large variability or insufficient animal number. Consistent with previous findings in *Abcc6^-/-^* and *Enpp1^asj/asj^* mice, dosing with recombinant ENPP1-Fc normalizes PPi levels rather than elevating PPi to supraphysiological levels presumably due to the wild-type copy of *Tnap* which regulates PPi to a physiological setpoint despite the much higher levels of ENPP1 activity.^35^^,^^40^ Although the IMA2a enzyme supplement normalized plasma PPi levels, this treatment did not change the CMD-relevant skeletal disease outcome in *Ank^KI/KI^* mice suggesting that reduced circulating PPi is not a major cause for CMD. The intracellular function of ANK, the local reduction of PPi in bone, and other yet undescribed dominant functions of CMD-mutant ANK are likely to play more important roles in CMD pathogenesis.

Systemic ENPP1-Fc enzyme is currently being assessed in clinical trials to support several indications involving low levels of PPi such as ENPP1 Deficiency (NCT04686175; NCT05734196; NCT06046820), ABCC6 Deficiency (NCT05030831), and calciphylaxis (NCT06283589). Human or mouse recombinant ENPP1-Fc protein has successfully decreased mortality and rescued pathological calcification in several ENPP1 and ABCC6 loss-of-function mouse models.^33-35^ ENPP1 uses ATP as substrate to generate PPi and AMP. The ATP release mechanisms involve vesicular exocytosis, plasma membrane F_1_/F_0_-ATP synthase, ATP-binding cassette (ABC) transporters, connexin hemichannels, pannexin channels, and ANK.^15^^,^^16^^,^^41^ Recently, ANK has been shown to release malate, succinate, citrate, and ATP, which is further converted into PPi and AMP by extracellular ENPP1 enzyme.^15^ Ank affects PPi levels mostly by transporting ATP rather than the release of appreciable amounts of PPi.^16^*Ank^KI/KI^* mice and ENPP1 deficient mice both exhibit reduced plasma PPi. However, unlike Enpp1 deficient mice, *Ank^KI/KI^* mice have wild-type copies of ENPP1. ENPP1 activity was unsurprisingly comparable in plasma from *Ank^+/+^* and *Ank^KI/KI^* mice that received vehicle treatment (Figure 1B). Activity of endogenous ENPP1 is orders of magnitude lower compared with the ENPP1 activity derived from IMA2a dosing, which this assay was optimized to detect. We showed that IMA2a treatment can elevate plasma PPi levels in *Ank^KI/KI^* mice even though the loss-of-function in mutant ANK prevents efficient transport of ATP, the substrate for conversion to PPi by ENPP1. We believe that this is because ATP release into circulation is co-regulated by ANK, ENPP1, and ATP binding cassette subfamily C member 6 (ABCC6). ENPP1 deficiency leads to almost complete absence of PPi in the systemic circulation in *Enpp1^KO/KO^* mice.^42^ ABCC6 mediates the release of ATP from hepatocytes into the circulation, which accounts for ~60% of plasma PPi.^38^ Similarly to the findings in our *Ank^KI/KI^* mice, *Abcc6^-/-^* mice also show low circulating PPi levels and dosing of these mice with recombinant ENPP1 (INZ-701) results in normalization of circulating PPi levels. Circulating recombinant ENPP1 enzyme may be able to access ATP substrate, not normally accessible to endogenous membrane-bound ENPP1, explaining why PPi increases despite the loss or reduced function of an ATP transporter (eg, ANK or ABCC6).

Our study further showed that IMA2a treatment serves to prevent the ectopic calcification of the joints in *Ank^KI/KI^* mice. To our knowledge, ectopic calcification around the foramen magnum or joint ankylosis has not been reported in patients with CMD. On the other hand, ANKH has been implicated in the maintenance of joint health. Ectopic calcifications of elbow, shoulder, and joints have been reported in several CMD patients showing radiographically ectopic calcifications of shoulders who were also suffering from calcium deposits in elbow and shoulder joints and hydroxyapatite crystals in the synovial fluid of shoulder and elbow joints, and episodic joint pain.^43^ Patients with familial chondrocalcinosis type 2 (CCAL2) carrying mutations in ANKH suffer from gout-like joint pain due to calcium pyrophosphate dihydrate (CPPD) crystal deposits.^44^ A missense mutation in ANKH (L244S) leads to mental retardation, spinal ankylosis, and periarticular calcifications of small joints in homozygous patients and a mild arthropathy in heterozygous individuals.^45^ Ankylosing spondylitis, genetically linked to ANKH, is characterized by chronic joint and entheseal inflammation, and ankylosis of axial and peripheral joints.^46^ The same eight-base pair repeat polymorphism in the 5' non-coding region of ANKH (ANKH-OR) has been reported as a genetic marker associated with temporomandibular joint closed lock.^47^ Ectopic calcification is regulated by the concentration of PPi. An excess of PPi can lead to CPPD formation, whereas reduced PPi levels can result in hydroxyapatite (HA) deposition. We have previously reported that ectopic calcification in *Ank^KI/KI^* mice is due to HA deposits, which is consistent with reduced concentrations of the circulating and local PPi.^22^ Findings from our current study suggest that ENPP1 enzyme replacement is beneficial for the prevention of ectopic calcification by excessive HA deposits on joints caused by low circulating PPi concentrations.

In *Ank^KI/KI^* mice, we observed calcified nodules adjacent to the foramen magnum. It remains unknown whether this calcific mass, even if it exists in patients, would contribute to any symptoms related to CMD. Although rare, calcifying tumors in the foramen magnum have been reported such as benign calcifying pseudoneoplasm of neuraxis (CAPNON) and meningioma. CAPNON lesions that impinge on critical neural structures may lead to symptoms such as pain, weakness, or seizures.^48^^,^^49^ One case report described a patient with foramen magnum meningioma with excessive calcification who experienced headaches and dizziness.^50^ In our study, we found a low positive correlation (correlation coefficient *r* = 0.3) between the volume of calcification at the foramen magnum and ENPP1 activity measured at the endpoint of *Ank^KI/KI^* mice receiving IMA2a. The lack of strong correlation may be due to one-time assessment. A longitudinal monitoring of ENPP1 activity during the treatment period of IMA2a may provide a better correlation with the volume of calcified nodules in *Ank^KI/KI^* mice. Results of this study suggest that ENPP1 enzyme replacement may potentially be considered for reduction of calcified masses at the foramen magnum in patients with CAPNON, especially since surgical treatment of these lesions is risky because of the complex anatomy. More studies will be required.

In summary, we have shown that with this current regimen IMA2a does not rescue the CMD phenotype in *Ank^KI/KI^* mice. This proof-of-principle study is significant because it helps to clarify the role of plasma PPi in the pathogenesis of CMD. Reduced circulating PPi is not a major factor for CMD pathogenesis. This study also revealed other potential applications for ENPP1 enzyme replacement such as for treating ectopic calcifications associated with reduced PPi levels.

## Supplementary Material

Supplemental_data_2024_07_11_ziae103

## Data Availability

Data available on request from the authors.
