# Supplementary material for: ENPP1 enzyme replacement therapy improves ectopic calcification but does not rescue skeletal phenotype in a mouse model for craniometaphyseal dysplasia
Source: JBMR Plus. 2024 Aug 8;8(9):ziae103. doi: 10.1093/jbmrpl/ziae103 (PMC11334334; doi:10.1093/jbmrpl/ziae103)
Supplement: Supplemental_data_2024_07_11_ziae103 [file supplemental_data_2024_07_11_ziae103.pdf]

Supplemental File

ENPP1 enzyme replacement therapy improves ectopic calcification but does not rescue skeletal phenotype in a mouse model for craniometaphyseal dysplasia

Ernst J. Reichenberger[1], Kevin O'Brien[2], Ayano Hatori[3], Thomas Carpenter[4], Koen van de Wetering[5], Lisa Flaman[2], Jennifer Howe[2], Daniel Ortiz[2], Yves Sabbagh[2], I-Ping Chen[1,3]

[1]Center for Regenerative Medicine and Skeletal Development, Department of Reconstructive Sciences, School of Dental Medicine, University of Connecticut Health, Farmington, Connecticut 06030, USA; [2] Research and Development, Inozyme Pharma, Boston, MA 02210, USA; [3]Department of Endodontology, School of Dental Medicine, University of Connecticut Health, Farmington, CT 06030, USA; [4]Department of Pediatrics (Endocrinology), Yale University School of Medicine, New Haven, CT 06520, USA; [5]Department of Dermatology and Cutaneous Biology, Jefferson Institute of Molecular Medicine and PXE International Center of Excellence in Research and Clinical Care, Sidney Kimmel Medical College, Thomas Jefferson University, Philadelphia, PA 19144, USA

Running title: Effects of ENPP1 on CMD

e-mail address of authors:
reichenberger@uchc.edu, kevin.obrien@inozyme.com, hatori@uchc.edu, thomas.carpenter@yale.edu, jan.vandewetering@jefferson.edu, lisa.flaman@inozyme.com, jennifer.howe@inozyme.com, daniel.ortiz@inozyme.com, yves.sabbagh@inozyme.com, ipchen@uchc.edu

Corresponding Author:

I-Ping Chen, DDS, PhD
Professor
Department of Endodontology
University of Connecticut Health
263 Farmington Avenue
Farmington, CT 06030-3705
Tel: 860-679-1030
Fax: 860-679-2910
email: ipchen@uchc.edu

Yves Sabbagh, PhD
Senior VP/Chief Scientific Officer
Inozyme Pharma, Inc.
321 Summer Street
Boston, MA 02210
Tel: 857.233.0115
Email: yves.sabbagh@inozyme.com

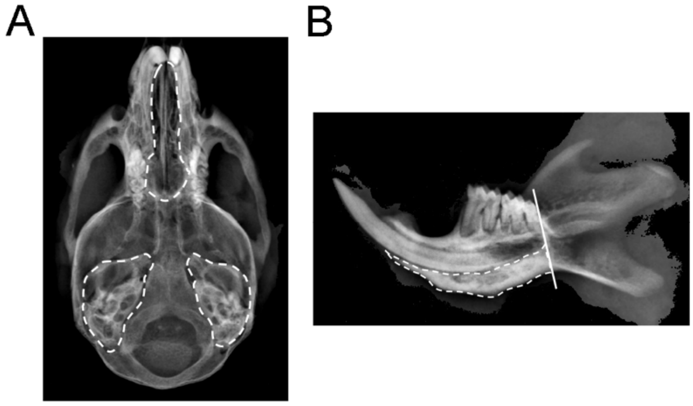

Supplemental Fig. S1. Radiographic data analysis presented in Fig. 2 B. **A**) Area (circled with white dashed line) selected to measure the intensity of gray value for radiographic opacity; **B**) Surface area (circled with white dashed line) measured to present the increased jawbone mass in *Ank*$^{KI/KI}$ mice.

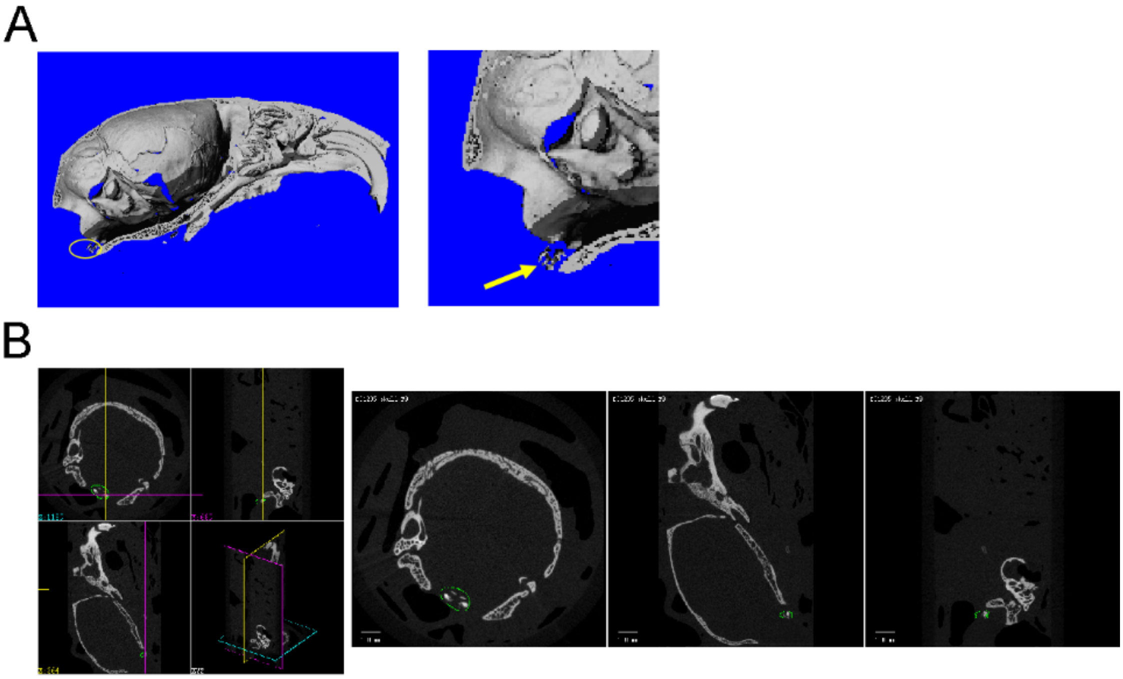

Supplemental Fig. S2. Localization of ectopic calcifications at the level of the foramen magnum in $Ank^{KI/KI}$ mice. **A)** Internal view of a representative 3-D skull images of an $Ank^{KI/KI}$ mouse that received vehicle treatment. The calcified mass is indicated by a yellow circle (left panel) and a yellow arrow in the magnified image (right panel). **B**) Cross-sectional slices of the $Ank^{KI/KI}$ skull at XY, YZ, XZ dimensions indicating the position of the calcified mass (green circles).

Supplemental Table S1: Primer sequences for genotype and sex determination

Names	Sequences
Ank-forward	5’-GCTAAGCTTCCATACTTACCCGTCTGC-3’
Ank-reverse	5’-CCTGCCCCTTACCTGGCACTG-3’
Sry-forward	5’-TGGGACTGGTGACAATTGTC-3’
Sry-reverse	5’-GAGTACAGGTGTGCAGCTCT-3’
IL3-forward	5’-GGGACTCCAAGCTTCAATCA-3’
IL3-reverse	5’-TGGAGGAGGAAGAAAAGCAA-3’

Supplemental Table S2: Results of 2-way ANOVA analyses in this study.

Factors	Sex	Groups	Interaction
Fig. 1B: ENPP1 activity	ns	**** $p < 0.0001$	ns
Fig. 1C: Plasma PPi conc.	ns	*** $p < 0.001$	ns
Fig. 2A: Intensity of gray value	ns	**** $p < 0.0001$	ns
Fig. 2B: Surface area of mandibles	ns	**** $p < 0.0001$	ns
Fig. 2C: Metaphyseal flaring	ns	**** $p < 0.0001$	* $p < 0.05$
Fig. 3A: Magnum foramen length	ns	**** $p < 0.0001$	ns
Fig. 3A: Magnum foramen width	* $p < 0.05$	** $p < 0.01$	ns
Fig. 3B: Trabecular thickness	ns	ns	ns
Fig. 3B: Trabecular number	** $p < 0.01$	ns	ns
Fig. 3B: Periosteum perimeter	ns	**** $p < 0.0001$	ns
Fig. 3B: Endosteum perimeter	ns	**** $p < 0.0001$	ns
Fig. 3C: Mandibular bone volume	** $p < 0.01$	**** $p < 0.0001$	ns
Fig. 3C: Mandibular total volume	** $p < 0.01$	**** $p < 0.0001$	ns
